# First-Principles Studies on the Structural and Electronic Properties of As Clusters

**DOI:** 10.3390/ma11091596

**Published:** 2018-09-03

**Authors:** Jialin Yan, Jingjing Xia, Qinfang Zhang, Binwen Zhang, Baolin Wang

**Affiliations:** 1 School of Materials Science and Engineering, Yancheng Institute of Technology, Yancheng 224051, China; yanjl@ycit.edu.cn (J.Y.); zhangbw@ycit.edu.cn (B.Z.); 2 College of Education Science, Nantong University, Nantong 226019, China; xiajj@ycit.edu.cn; 3 School of Physical Science and Technology, Nanjing Normal University, Nanjing 210023, China

**Keywords:** atomic clusters, density functional theory, genetic algorithm

## Abstract

Based on the genetic algorithm (GA) incorporated with density functional theory (DFT) calculations, the structural and electronic properties of neutral and charged arsenic clusters As_n_ (*n* = 2–24) are investigated. The size-dependent physical properties of neutral clusters, such as the binding energy, HOMO-LUMO gap, and second difference of cluster energies, are discussed. The supercluster structures based on the As_8_ unit and As_2_ bridge are found to be dominant for the larger cluster As_n_ (*n* ≥ 8). Furthermore, the possible geometric structures of As_28_, As_38_, and As_180_ are predicted based on the growth pattern.

## 1. Introduction

In recent years, due to the fast development of nanotechnology, people are more interested in atomic clusters, which are composed of several to thousands of atoms, molecules, or ions through a physical or chemical bonding force [1]. Clusters can also be regarded as the transitional forms between atoms and bulk, and their fundamental properties depend vitally on the cluster size. Therefore, it is quite meaningful to study the structural and electronic properties of clusters using theoretical research, identifying their potential capacities for numerous applications. Arsenic has been widely applied in many fields such as semiconductors, optoelectronics, and biopharmaceutics [2,3,4,5,6,7,8,9,10,11]. Besides, vanadium doped phosphorus clusters, and pure and doped arsenic clusters have received a large amount of attention from both experimental and theoretical fields in recent years [12,13,14,15,16,17,18,19,20,21,22,23]. 

Experimentally, the study of As_n_ clusters has focused on small-sized clusters with *n* ≤ 5 [12,13,14,15,16,17]. For example, Wang et al. [12] utilized high-resolution photoelectron spectroscopy to study the electronic vibration and spin orbit of As_4_. Bennett et al. [13] have measured the appearance potentials and ion translational energies for the As_1_, As_2_, and As_3_ ions formed by the dissociative resonance capture of As_4_. Lippa et al. [14] have probed the electron affinities of As_n_ (*n* = 1–5) using photoelectron spectra in 1998. Brumbach and Rosenblatt [15] have investigated the vibrational modes of As_4_ with Raman spectroscopy. Yonezo [16] designed a high-temperature nozzle assembly for gas-electron diffraction to determine the structure of As_4_. Jeffrey et al. [17] measured the ionization potentials (IPs) for As_n_ (*n* = 1–5) using gas-phase charge-transfer reactions. No experimental data are available for As_n_ with *n* ≥ 6 right now.

In the theoretical aspect, Zhao et al. [18] and Bai et al. [21] studied the structures, thermochemistry, and electron affinities of As_n_ (*n* = 1–16) and their anions. Their results showed that the even-numbered neutral As_n_ species are more stable than the odd-numbered clusters, but the even-numbered anionic As_n_ species are less stable than the odd-numbered species. Liang et al. [19] probed the electronic structure and property of neutral and charged arsenic clusters As_n_^(+1,0,−1)^ (*n* = 2–8). At the B3LYP/6-311+G(d) theoretical level, Guo [20] investigated the geometries and energies for neutral and charged As_n_ (*n* = 2–15) clusters, and reported their relative stability, ionization potential, and electron affinity. Baruah et al. [23] using a generalized gradient approximation (GGA) to explore the geometry, vibrational modes, and polarizabilities, as well as the infrared and Raman spectra of fullerene-like arsenic cages with *n* = 4, 8, 20, 28, 32, 36, and 60. Zhao et al. [22] investigated the structures and electronic properties of As_n_ clusters with even-numbered As_n_ (*n* = 6–28) using density functional theory (DFT) with the Perdew–Burke–Ernzerhof functional and a doubled numerical basis set with d-polarization functions(PBE/DND) scheme and found that the supercluster structures based on As_4_, As_6_, and As_8_ units, and the As_2_ bridge were dominant for the larger As_n_ with *n* ≥ 14. 

Although many theoretical works have been performed on the As_n_ clusters, all the results are always most of the artificial speculation studies to investigate the structure of clusters in certain symmetries. In this work, we have performed a genetic algorithm (GA) incorporated with density functional theory (DFT) calculations to explore the structures and electronic properties of As_n_ (*n* = 2–24) neutral and charged clusters. After we have determined the growth pattern of As_n_ clusters, the possible geometric structures of As_28_, As_38_, and As_180_ are predicted based on the growth pattern. We also discussed the size-dependent physical properties of neutral clusters such as the binding energy, HOMO-LUMO gap and second difference of cluster energies.

## 2. Computational Methodology

In order to search the global minimum structures of As_n_ clusters, we combined a genetic algorithm (GA) simulation with local optimization at the Dmol^3^ level [24,25,26,27,28,29]. The fundamental aim in GA is to divide the potential-energy surface (PES) into a number of regions and find the locally stable isomers in each region. In the GA program, we generated 15 As_n_ (*n* = 3–12) and 20 As_n_ (*n* = 13–24) initial populations to ensure that we could find the local minimum. Any population can be chosen as parents to generate their child cluster according to a crossover operation. In addition, there was a possibility of a 30% mutation rate for a single parent to produce child alone. The child cluster was optimized with Dmol^3^, and then compared with its parent in energy. The child with lower energy replaced its high-energy parent. The whole process of genetic algorithm with 2000 iterations was to ensure we got the lowest energy structure.

The optimization of As_n_ (*n* = 2–24) clusters was performed using DFT with the Perdew–Burke–Ernzerhof (PBE) [24] exchange-correlation functional and an all-electron basis set of the double-numerical-plus-d-polarization (DND) type, as implemented in the Dmol^3^ [25] package. A self-consistent field calculation kept the accuracy with an energy convergence for 10^−6^ *a.u.*, and the forces for 2 × 10^−3^ *a.u.* There was no symmetry restriction for geometry optimization.

For each As_n_ (*n* = 2–24) cluster, we saved ten energetically lower isomer structures for further electronic structure calculations, which were performed using the Vienna Ab-initio Simulation Package (*VASP*) codes. The Kohn–Sham equations were solved variationally in a plane wave basis set using the projector-augmented-wave (PAW) method. The exchange-correlation energy was described by the functional of Perdew, Burke, and Ernzerhof (PBE) based on the generalized gradient approximation (GGA). The energy cutoff was set to be 400 eV and the vacuum space was set to be at least 14 Å to separate the interactions between the neighboring slabs. Only the Gamma k-point was used to sample the Brillouin zone for the geometry and electronic structure calculations. All structures were fully relaxed by Gaussian smearing and electronic structure calculations by tetrahedron smearing method until the convergence criteria (with the force less than 0.02 eV/Å and the energy less than 10^−5^ eV). Although each structure of As_n_ (*n* = 2–24) cluster was further optimized by *VASP*, the energy sequencing of each cluster was basically unchanged.

## 3. Results and Discussions

### 3.1. As_n_ (n = *2–8*) Clusters

**Figure 1:**
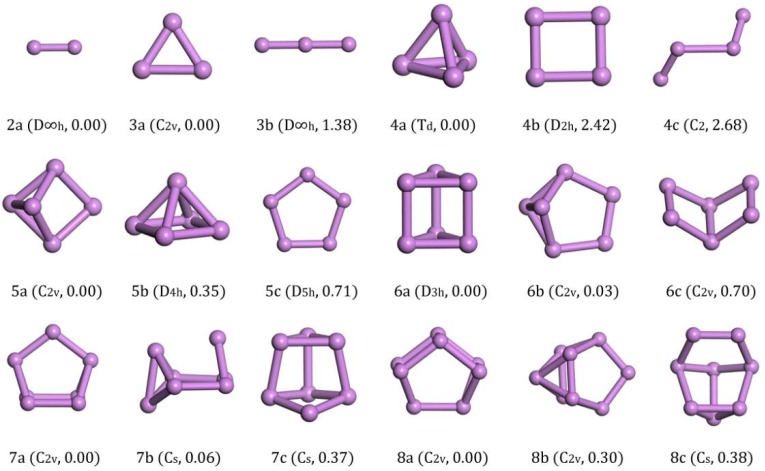
Lowest-energy and isomorphic structures for As_n_ (*n* = 2–8) clusters. The relative total energies are in eV.

The bond length of an As_2_ cluster (2a in Figure 1) is 2.103 Å through experimental measurement [30]. In our calculation the distance between the As atoms was 2.118 Å, which is closer to the experimental data compared with the 2.142 Å calculated using PBE/DND methods [22]. 

For the As_3_ cluster, the energy of the structure with C_2v_ symmetry (3a in Figure 1) was the global minimum. It was an isosceles triangle structure with a top angle of 65.14° and side length of 2.325 Å. It was energetically lower than the linear chain structure with D∞h symmetry (3b in Figure 1), in which structure, the bond length was 2.204 Å.

The ground-state structure of As_4_ with T_d_ symmetry (4a in Figure 1) was a regular tetrahedron, which was consistent with the previous reports [18,19,20]. Its energy was much lower than other isomorphic configurations. The energy of the rectangular structure with D_2h_ symmetry (4b in Figure 1) was 0.605 eV/atom higher than that of the ground state and the chain structure with C_2_ symmetry (4c in Figure 1) was energetically higher than the rectangular structure.

The lowest energy structure of As_5_ had C_2v_ symmetry (5a in Figure 1) and it may be considered as adding an atom in the cross section of a dihedral formed by four atoms. It had 0.07 eV/atom less energy than the rectangular pyramid structure with D_4h_ symmetry (5b in Figure 1) and 0.142 eV/atom less than the planar structure with D_5h_ symmetry (5c in Figure 1).

The trigonal prism with D_3h_ symmetry (6a in Figure 1) was the ground-state structure for As_6_ and was only 0.005 eV/atom lower than the benzvalene type with C_2v_ symmetry (6b in Figure 1) and 0.117 eV/atom lower than the dihedral angle structure of six atoms with C_2v_ symmetry (6c in Figure 1). The side length was 2.522 Å and the edge length was 2.559 Å. Our ground state was consistent with the result calculated by B3LYP/6-311+G(d) [20] or PBE/DND methods [22]. However, the structure 6b in Figure 1 was found to be the lowest energy by Liang [19] using MP2(full)/g-31G(d) methods and Bai [21] using B3LYP/DZP++ methods. As the outer shell structure of As is 3*s*^2^3*p*^3^, we think our results are reasonable as the completely three-coordination structure (6a in Figure 1) must be more stable than the structure with two two-coordinations (6b in Figure 1).

In the case of As_7_, the ground-state structure with C_2_v symmetry (7a in Figure 1) could be derived from the trigonal prism of As_6_ by edge-capping with an additional As atom. This low-energy structure is also predicted in Refs. [19,20,21]. Its energy was lower than the structure with Cs symmetry (7b in Figure 1) by 0.008 eV/atom and the structure with Cs symmetry (7c in Figure 1) by 0.053 eV/atom. 

The wedge-like structure that looks like a cage with C_2v_ symmetry was obtained as the lowest energy structure for As_8_, as seen from 8a in Figure 1. It was energetically lower than the structure with C_2v_ symmetry (8b in Figure 1) by 0.038 eV/atom and the structure with Cs symmetry (8c in Figure 1) by 0.048 eV/atom. We also checked the cage structure with O_h_ symmetry cut from the bulk phase reported by Baruah [23], and we found that it was 0.64 eV energy higher than our ground state structure.

After analysis of the ground structures of As_n_ (*n* = 2–8) clusters, we find that As_2_ was a one-dimensional bridge, As_3_ was a two-dimensional isosceles triangle and As_4_ became a three-dimensional tetrahedron. When *n* was larger than 3, the two-dimensional cluster structure, such as 4b and 5c in Figure 1, sorts more and more backward energetically. We can conclude that the structure of small As clusters tends towards a three-dimensional cage structure and it was not stable for a 2-D planar structure.

### 3.2. As_n_ (n = *9–18*) Clusters

**Figure 2:**
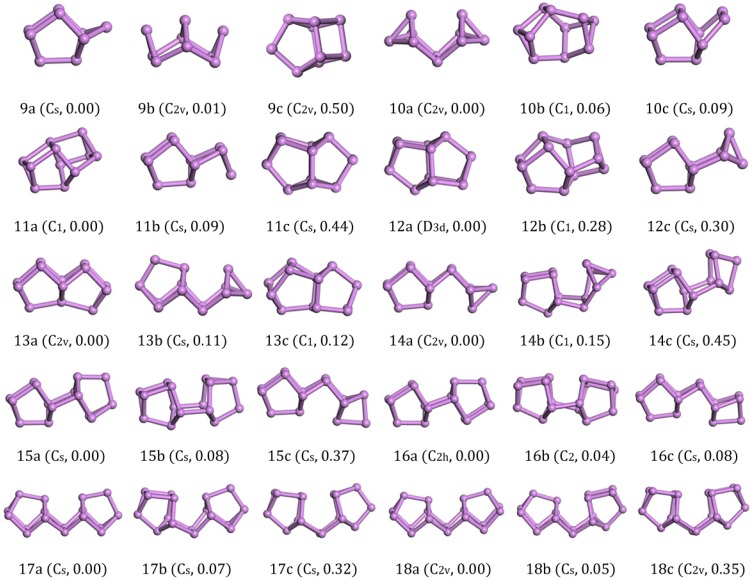
Lowest-energy and isomorphic structures for As_n_ (*n* = 9–18) clusters. The relative total energies are in eV.

The ground-state structure of As_9_ with C_s_ symmetry (9a in Figure 2) could be regarded as being derived from a cage-like As_8_ structure by attaching an As atom at one side. The isomers (C_2v_) with a higher symmetry (9b,c in Figure 2) were less stable based on our *VASP* calculations. It also hinted to us that the ground structure of As_8_ might be a stable cluster with a magic number. From further calculations, we found that the cage-like As_8_ structures unit served as the primary building unit for forming the As clusters with larger sizes. 

The lowest-energy structure (C_2v_) of As_10_ consistd of the cage structure of 8a in Figure 1 that was edge-capped by each As atom. After relaxation, the upper bond broke to form two four-atom cages and an As_2_ bridge. The structure 10b in Figure 2 was a new structure discovered by our GA global searching. The structure obtained by Zhao [22] is structure 10c in Figure 2, whose energy was higher than 10b in Figure 2 by 0.006 eV/atom and 10a in Figure 2 by 0.009 eV/atom.

The ground structure of As_11_ was 11a in Figure 2 which was formed on the base of As_10_ with an As atom added above the As_2_ dimer and linked with an As_8_ cage. It was more stable than the 11b in Figure 2 isomer with C_s_ symmetry by 0.008 eV/atom and 11c in Figure 2 isomer by 0.04 eV/atom with C_s_ symmetry in energy. 

For As_12_, the structure with D_3d_ symmetry was confirmed to be the lowest energy structure among all the structural candidates considered. The highly-symmetric structure was shaped of two As_8_ cages that share a four-atom plane. From another point of view, the ground-state structure of As_12_ was a layered structure of three layers of atoms (3 + 6 + 3). Such a nice structure was energetically lower than 12b in Figure 2 with C_1_ symmetry by 0.023 eV/atom and 12c in Figure 2 with C_s_ symmetry by 0.025 eV/atom. 

Viewing the ground-state structure of As_13_ carefully, we also found two As_8_ cages. Different from As_12_, the two cages jointly owned a three-atom plane. It had 0.008 eV/atom less energy than the structural 13b in Figure 2 (C_1_) and 0.009 eV/atom less than the structure 13c in Figure 2 (C_s_).

As shown in the picture, As_14_ with Cs symmetry could be considered to be composed of an As atom link to the three-atom plane on one side of the As_13_ (13a in Figure 2). The less stable isomer 14b was a distorted structure of 14a, which was energetically higher by 0.011eV/atom. The 14c was a structure without an As_2_ dimer bridge, it was linked by As_8_ cage with an As_6_ cage and had 0.032 eV/atom more energy than 14a in Figure 2.

The ground-state structure of As_15_ with C_s_ symmetry was composed of two connected cages (As_8_ and As_7_). The other two candidates 15b in Figure 2 with C_s_ symmetry and 15c in Figure 2 with C_s_ symmetry were less stable than the ground-state structure by 0.005 eV/atom and 0.025 eV/atom in energy, respectively.

An upward As_8_ cage and a downward As_8_ cage connected to form a new structure as the ground state of As_16_ (16a in Figure 2 with C_2h_ symmetry). It was more stable than the C_2_-symmetry isomer 16b in Figure 2 and the C_s_-symmetry isomer 16c in Figure 2 by 0.05 eV and 0.08 eV in energy. Although structural 16c in Figure 2 contained an As_8_ cage and an As_2_ bridge, the As_6_ cage in the structure led to the overall energy as being higher than other two isomers.

The lowest energy structure of As_17_ (17a in Figure 2) with C_s_ symmetry was built by As_8_ and As_7_ units with an As_2_ bridge in the middle. The two C_s_-symmetry isomorphic structure 17b,c in Figure 2 were also formed by the As_8_ and As_7_ units with different orientation connections. 

The ground-state of As_18_ (18a in Figure 2) with C_2v_ symmetry was formed by two identical As_8_ units and an As_2_ bridge in the middle. Our structure was exactly the same as that in Ref. [22]. Two slightly higher energy isomers (18b in Figure 2) with C_2h_ symmetry and 18c in Figure 2 with C_2v_ symmetry were combined by the same units as 18a in Figure 2 with different orientations, and they were energetically higher than 18a in Figure 2 by 0.05 eV and 0.35 eV energy. The calculations showed that the structure with As_8_ unit and As_2_ bridge in the middle was more stable than other cage structures. We also generated one As_18_ structure cut from bulk phase and the energy was 3.17 eV higher than the ground state. So, we think the chain structure with As_8_ units and an As_2_ bridge is much more important for middle-sized As_n_ clusters.

### 3.3. As_n_ (n = *19–24*) Clusters

**Figure 3:**
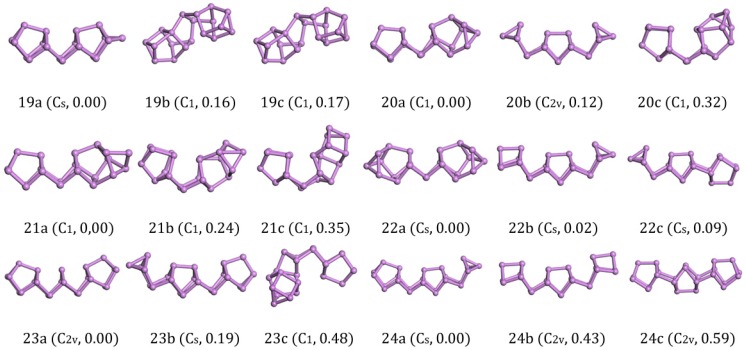
Lowest-energy and isomorphic structures for As_n_ (*n* = 19–24) clusters. The relative total energies are in eV.

The ground-state structure of As_19_ (19a in Figure 3) with C_s_ symmetry could be regarded as an As atom added into one side of As_18_. Therefore, As_19_ (19a in Figure 3) could be considered as the combination of As_8_-As_2_-As_8_-As_1_. The isomers of As_19_ (19b,c in Figure 3) with only C_1_ symmetry were both built up by two identical As_8_ units and an As_3_ bridge. Due to the distortion of the structures, they had 0.008 eV/atom and 0.009 eV/atom higher energy than 19a in Figure 3.

The ground-state structures of As_20_ was predicted to be C_1_ symmetry, as shown in 20a in Figure 3. It could be regarded as an As_8_ cage link with an As_10_ cage joined by an As_2_ bridge. As_20_ (20a in Figure 3) could be considered as the combination of As_8_-As_2_-As_10_. Zhao [22] predicted the optimal combinations for the As_20_ is super-clusters of As_4_-As_2_-As_8_-As_2_-As_4_. The energy of As_20_ (20a in Figure 3) we got based on the genetic algorithm was energetically lower than the structure 20b in Figure 3 with C_2v_ symmetry. A distorted structure 20c in Figure 3 originated from 20a in Figure 3 also appeared in our calculations. After the calculations with VASP, two isomers showed 0.006 eV/atom and 0.053 eV/atom higher energy than 20a in Figure 3. This result shows the super-clusters of As_4_-As_2_-As_8_-As_2_-As_4_ did not have much of an advantage.

For As_21_, the most stable structure (21a in Figure 3) could be regarded as an As atom link to the As_10_ cage of As_20_ (20a in Figure 3). Two other isomers (21b,c in Figure 3) were constituted by an As_8_ cage and an irregular As_11_ structure linked with an As_2_ bridge. They were energetically higher than 21a in Figure 3 by 0.011 eV/atom and 0.017 eV/atom.

Rather than simply increasing the number of atoms on the edge, the ground-state structure of As_22_ with C_s_ symmetry are formed with two symmetrical As_10_ cages in the As_20_ units and an As_2_ bridge in the middle. The isomers were two kinds of super-clusters (22b in Figure 3, As_6_-As_2_-As_8_-As_2_-As_4_ and 22c in Figure 3, As_4_-As_2_-As_8_-As_8_), and they were stretched by more units compared to the ground-state structures (22a in Figure 3). Although we could find stable As_8_ and As_4_ units in isomers, they each had 0.02 eV and 0.09 eV higher energy than 22a in Figure 3. According to previous findings, it can be found that the second lower energy As_10_ cage 10b in Figure 2 will be favorable if the structure is composed by an As_8_ unit connected with an As_2_ bridge.

The ground-state structures of As_23_ (23a in Figure 3) with C_2v_ symmetry seemed to be four As_8_ cages linked to each other to share the three-atom plane, and the bottom edge of the middle two cages were broken. At the same time, we could also regard 23a in Figure 3 as a super-cluster of As_8_-As_2_-As_3_-As_2_-As_8_. The other two candidates, 23b in Figure 3 with C_s_ symmetry and 23c in Figure 3 with C_1_ symmetry, were less stable than the structure 23a in Figure 3 by 0.008 eV/atom and 0.021 eV/atom in energy, respectively.

The lowest energy structure of As_24_ with C_s_ symmetry (24a in Figure 3) was built by three units (an As_4_ cage and two identical As_8_ cages), connecting the neighboring structure with an As_2_ bridge. Zhao [22] considered that the optimal combinations of the super-clusters As_24_ are As_6_-As_2_-As_8_-As_2_-As_6_ (24b in Figure 3) with C_2v_ symmetry. DFT calculations show that the ground-state structure of As_24_ we get based on the genetic algorithm weare the combinations of As_8_-As_2_-As_8_-As_2_-As_4_, which was energetically lower than the structure 24b in Figure 3 by 0.018 eV/atom. Besides, we also gained another high symmetric structure with C_2v_ symmetry (24c in Figure 3) that was constituted by three As_8_ units. However, its energy was 0.025 eV/atom higher than the ground state structure (24a in Figure 3). We could realize from this result that the lowest energy structures of larger As clusters not only have the combination of As_8_ units but also needed an As_2_ bridge in the middle of adjacent units.

### 3.4. As_n_ (n = *2–24*) Charged Clusters

We also studied the ground structures of As_n_ (*n* = 2–24) charged clusters. Theoretically, it was easy to simulate a cationic or anionic cluster by adjusting the total electrons from the neutral cluster. From Figures S2 and S3, we could know the lowest energy structures of charged clusters (*n* = 2–4) were almost the same as with neutral cases. For As_8_ clusters, 8a in Figure 1 structures were quite stable even in cationic or anionic cases and it was the cluster with the magic number. For other As_n_ (*n* < 16) clusters, the isomers changed the energy sequence as the system changed the electron numbers. It was interesting to find that the structures for As_n_ (15 < *n* < 24) clusters were quite stable whatever attachment of extra electron to the neutral or losing of an electron from the neutral cluster.

### 3.5. As_n_ (n = *28, 38, 40, 180*) Clusters

**Figure 4:**
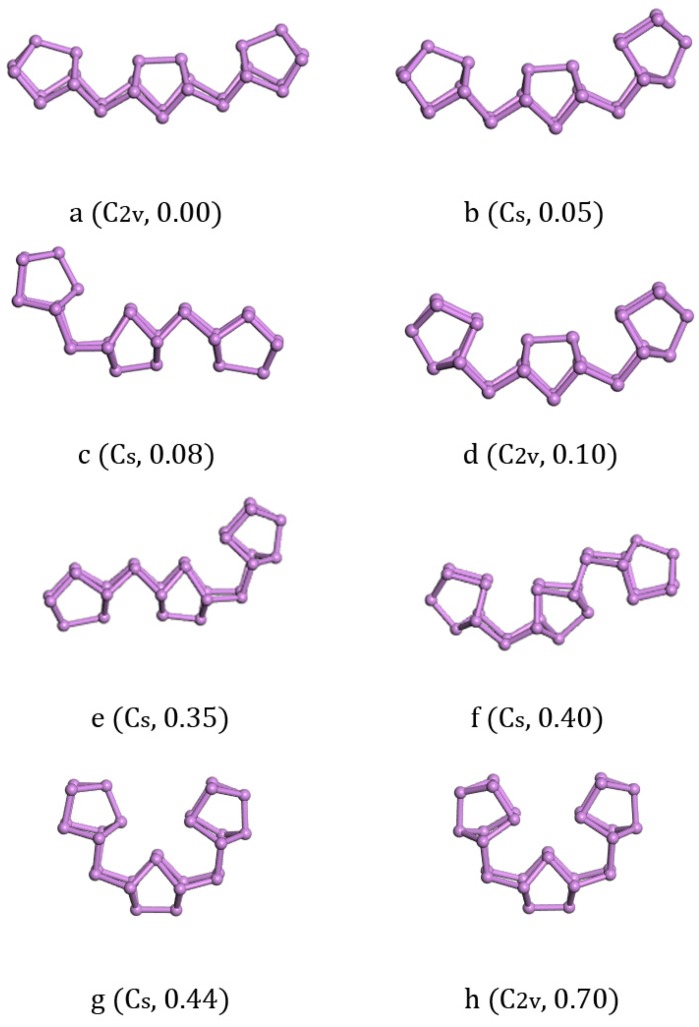
Lowest-energy and isomorphic structures for As_28_ clusters. The relative total energies in eV.

With the increase of cluster size, it was more and more difficult to exhaust all possible local minimum structures. We tried to study the larger clusters As_28_, As_38_, and As_40_ based on the above findings. The structural size evolution and electronic properties of arsenic clusters indicated that the clusters combined by an As_2_ bridge and an As_8_ cage had lower energy than their isomers and showed more stability in each local size-dependent range. Here we have to emphasize that As_4_ and As_6_ units were not dominant for the larger As_n_ cluster, which was different from Zhao’s result [22]. Furthermore, different sizes of fullerene cage structure isomers were also calculated to compare with our ground state structures in energy, and their energies were far more than units-linked one-dimensional structures. Given this understanding, we constructed As_28_ as As_8_-As_2_-As_8_-As_2_-As_8_ and As_38_ as As_8_-As_2_-As_8_-As_2_-As_8_-As_2_-As_8_ in all possible ways. The structures we calculated of As_28_ are listed along with the increase of energy in Figure 4. The lowest energy structure of As_28_ with C_2v_ symmetry (Figure 4a) had 0.05 eV less energy than the structure with C_s_ symmetry (Figure 4b) and 0.10 eV less than the structure with C_2v_ symmetry (Figure 4c). In addition to considering structure growth in the one-dimensional direction, we also calculated the longitudinal growth mode. Three isomers Figure 4c,e,f were all with C_s_ symmetry and energetically higher than the lowest energy structure a with 0.08 eV, 0.35 eV, and 0.40 eV, respectively. Besides, the other two semi-ring isomers Figure 4g,h had 0.44 eV and 0.70 eV energy higher than the ground state structure. Compared with the bulk truncated structure of As_28_, the lowest energy structure of As_28_ was 0.16 eV/atom lower.

**Figure 5:**
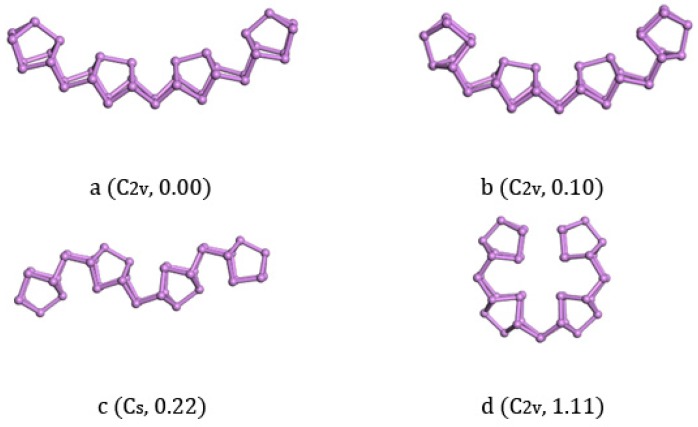
Lowest-energy and isomorphic structures for As_38_ clusters. The relative total energies are in eV.

The lowest energy structure of As_38_ with C_2v_ symmetry (Figure 5a) and its isomers are listed in Figure 5. We could find that the structure of Figure 5a was a continuation of As_8_, As_18_, and As_28_ clusters, and all of them were constructed with As_8_ cages in the same direction with As_2_ bridges. The structural isomers Figure 5b with C_2v_ symmetry and Figure 5c with C_2h_ symmetry could be regarded as one-dimensional chain structures, the same as Figure 5a. Their respective energies were 0.10 eV and 0.22 eV higher than Figure 5a. The C_2v_ isomer Figure 5d could be regarded as a two-dimensional structure that has four As_8_ cages held in four directions and all of them point to the center. The energy of this structure was highest in our calculation with 1.11 eV higher energy than a. Compared with the bulk truncated structure of As_38_, the lowest energy structure of As_38_ was 0.156 eV/atom lower. We could find that the structures did not change previous growth tendencies even with increasing the size of the clusters.

**Figure 6:**
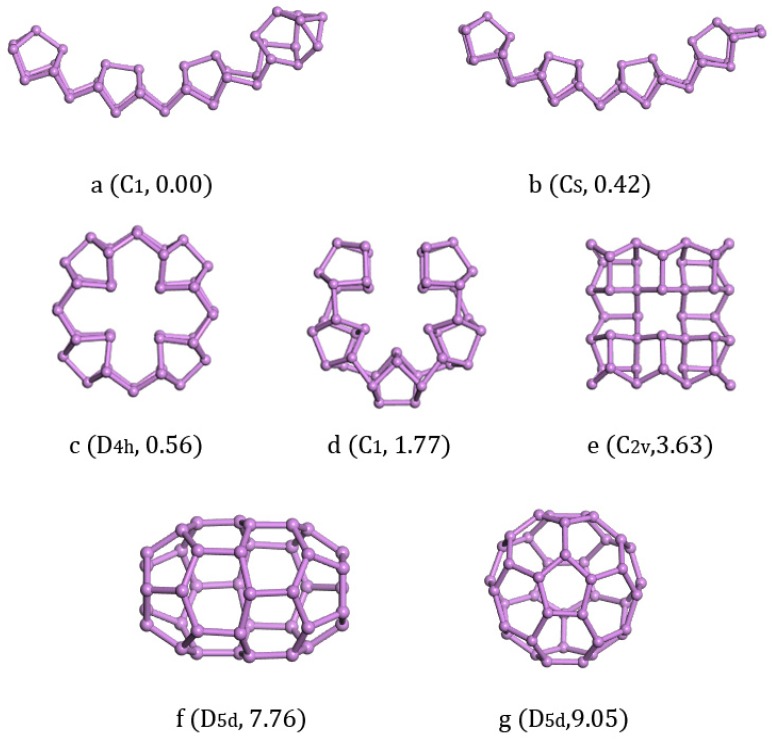
Lowest-energy and isomorphic structures for As_40_ clusters. The relative total energies in are eV.

Considering that As_40_ can form ring and fullerene cage structures, we also studied the structures of As_40_. The structures we calculated of As_40_ are listed along with the increase of energy in Figure 6. We found that the lowest energy structure of As_40_ with C_1_ symmetry (Figure 6a) and its isomers Figure 6b (0.42 eV energy higher) with C_s_ symmetry both were one-dimensional chain structures. Three isomers Figure 6c,d,e could all be regarded as two-dimensional structures and energetically higher than the lowest energy structure a with 0.014 eV/atom, 0.044 eV/atom, and 0.09 eV/atom, respectively. Seemingly stable three-dimensional fullerene cage isomers Figure 6f with D_5d_ symmetry and Figure 6g with D_5d_ symmetry were 0.194 eV/atom and 0.226 eV/atom higher in energy than the lowest energy structure.

**Figure 7:**
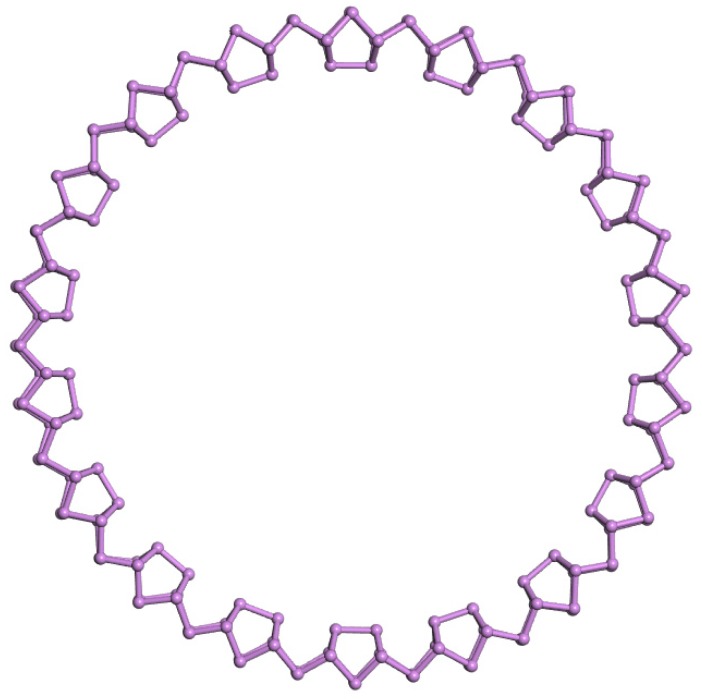
Possible structure for As_180_ cluster.

Based on the finding above, we could construct the ring structure of an As_180_ cluster based on the As_8_ units and As_2_ bridge, which is shown in Figure 7. The HOMO-LUMO gap of As_180_ was 1.868 eV and the binding energy per atom was −2.901 eV.

### 3.6. Electronic Properties of As_n_ Clusters

**Figure 8:**
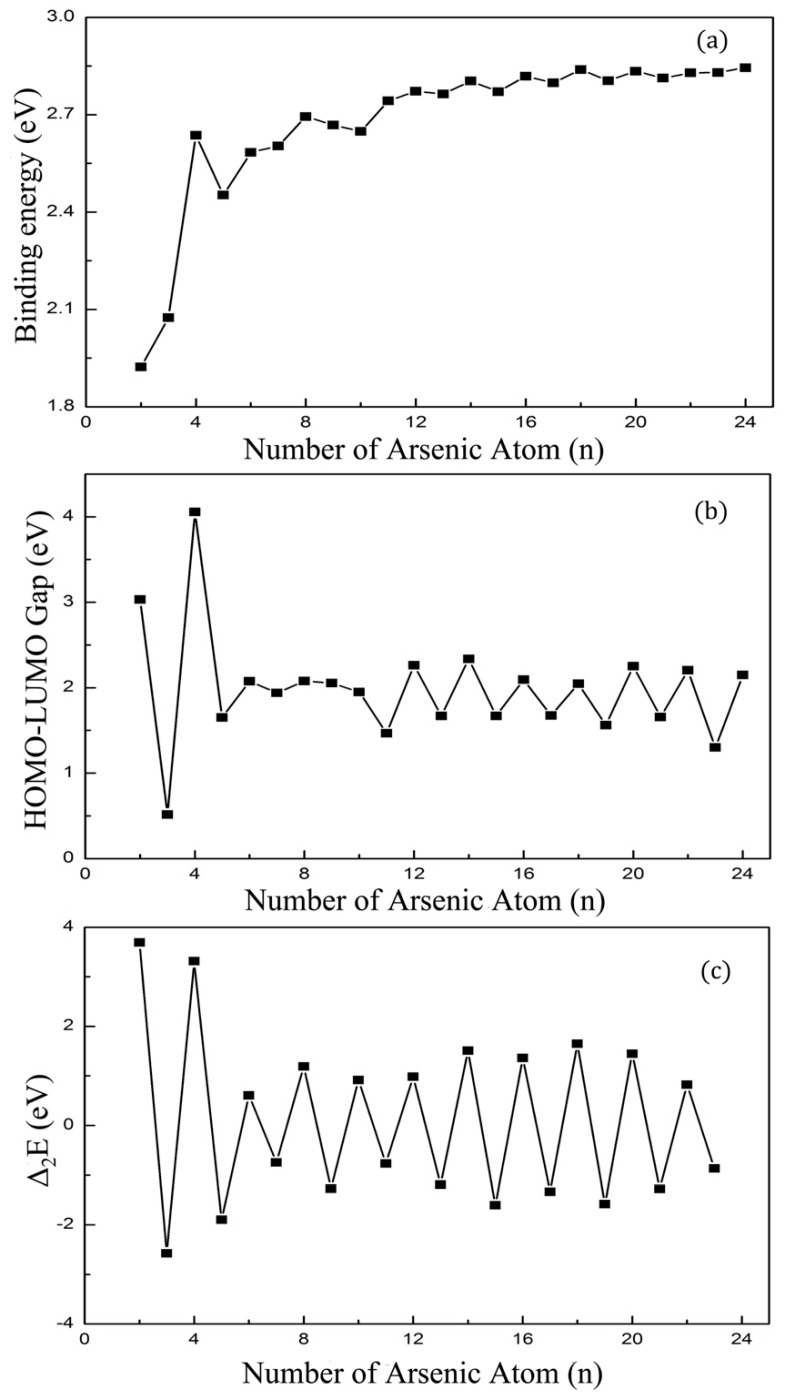
(**a**) Average binding energies (**b**) HOMO-LUMO Gaps and (**c**) second-order difference of cluster energies of As_n_ clusters.

The binding energy per atom for the ground states of As_n_ (*n* = 2–24) clusters are shown in Figure 8a. In the size range of *n* = 12–24, the binding energy increased smoothly with weak odd-even oscillation properties. This result can be related to the evolution of the ground-state from cage-like structure to cage-link structure at *n* = 12. Besides, the binding energy of As_8_ was a peak value in the small size of *n* = 3–11, and this suggests the ground structure of As_8_ would be a vital growth unit in larger structures. Our next calculation also proved the conjecture.

In Figure 8b, we present the energy gaps between the highest occupied molecular orbital (HOMO) and lowest unoccupied molecular orbital (LUMO) for the lowest-energy state of As_n_ (*n* = 2–24) clusters. As is known, the cluster with the larger energy gap is more stable and easier to prepare. As the largest energy gap of As_4_ is 4.06 eV, it is the most prominent species in arsenic vapor, leading to a number of experimental and theoretical studies on As_4_ clusters. Although the gaps of As_n_ clusters from *n* = 5–10 change smoothly, we also observe the gap of As_8_ is highest locally, which points out that the stability of As_8_ was higher than the neighboring cluster. The HOMO-LUMO gap was higher for As_n_ (*n* = 4, 6, 8, 12, 14, 16, 18, 20, 22, and 24) than their adjacent structures. The atoms in these even-numbered sequences were all three-coordination and eight-electron structure. The odd-numbered clusters were unable to achieve this condition, so they were less stable than the odd-numbered species. 

In clusters physics, the second-order difference of cluster energy is a more sensitive datum to reflect the stability of clusters. We plotted the second-order difference of cluster energies defined by Δ^2^*E* = E(*n* + 1) + E(*n* − 1) − 2E(*n*). Figure 8c describes how the second-order differential energy changed with the increase of atom number and it shows good odd–even oscillation properties. The second-order difference of cluster energies of even-numbered clusters were all higher than their adjacent odd-numbered clusters. Therefore, we could draw the conclusion that even-numbered clusters were more stable than their neighboring odd-numbered clusters. Above all peaks for Δ^2^*E*, three local maximum peaks were found at *n* = 4, 8, and 18, where As_n_ (*n* = 4, 8, and 18) clusters were chemically stable.

## 4. Conclusions

We have adopted the genetic algorithm and all-electron DFT calculations to systematically study the structures and electronic properties of As_n_ (*n* = 2–24). The ground-state structures of As_n_ clusters change from two to three dimensional after *n* = 3. Arsenic clusters followed a structural growth pattern starting from *n* = 8 and the structures of As_n_ (*n* = 9–24) clusters could all be regarded as evolving from the ground state structure of an As_8_ unit and an As_2_ bridge. The binding energy of As_n_ (*n* = 2–24) clusters had a periodic step-like behavior, and the size-dependent HOMO-LUMO gap and second-order difference of cluster energies exhibited obvious even–odd alternations with several magic numbers. Based on the growth pattern concluded from small As_n_ clusters, the possible superstructures of As_28_, As_38,_ As_40_, and As_180_ were discussed.

## Supplementary Materials

The following are available online at http://www.mdpi.com/1996-1944/11/9/1596/s1, Figure S1: Low energy isomers of As*_n_* (*n* = 2–24) neutral clusters, Figure S2: Low energy isomers of As^+^*_n_* (*n* = 2–24) cationic clusters, Figure S3: Low energy isomers of As^-^*_n_* (*n* = 2–24) anionic clusters.
