# Supplementary material for: First-Principles Studies on the Structural and Electronic Properties of As Clusters
Source: Materials (Basel). 2018 Sep 3;11(9):1596. doi: 10.3390/ma11091596 (PMC6163391; doi:10.3390/ma11091596)
Supplement: Supplementary file 1 [file materials-11-01596-s001.docx]

First-Principles Studies on the Structural and Electronic Properties of As Clusters

**Jialin Yan ^1,2,3^, Jingjing Xia ^2^, Qinfang Zhang ^1,^*, Binwen Zhang ^1^, and Baolin Wang ^3,^***

^1^ School of Materials Science and Engineering, Yancheng Institute of Technology, Yancheng 224051, P.R. China

^2^ College of Education Science，Nantong University, Nantong, 226019, P.R. China

^3^ School of Physical Science and Technology, Nanjing Normal University, Nanjing 210023, P.R. China


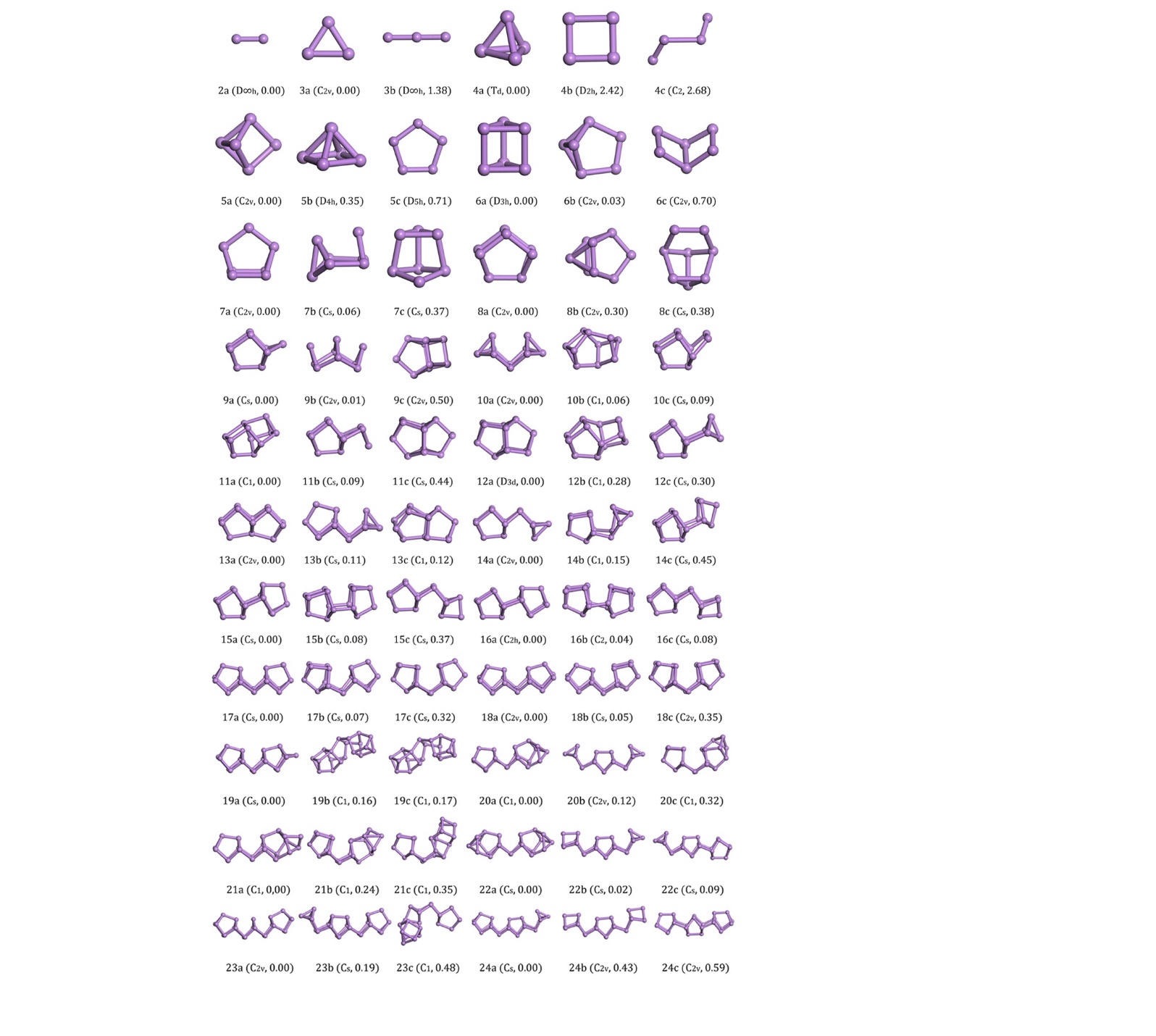


**Figure S1**. Low energy isomers of As*_n_* (*n*=2–24) neutral clusters.


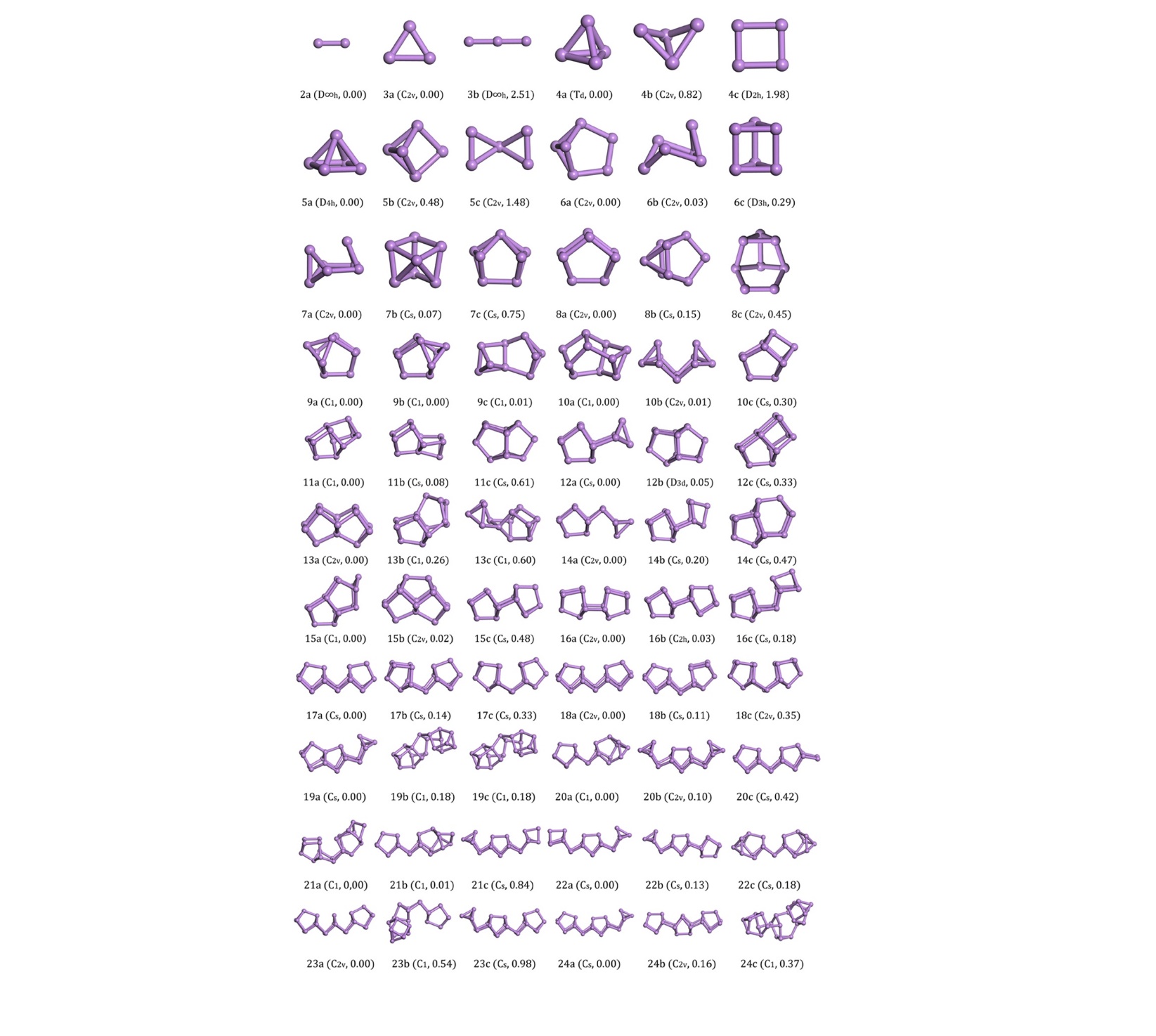


**Figure S2.** Low energy isomers of As^+^*_n_* (*n*=2–24) cationic clusters.


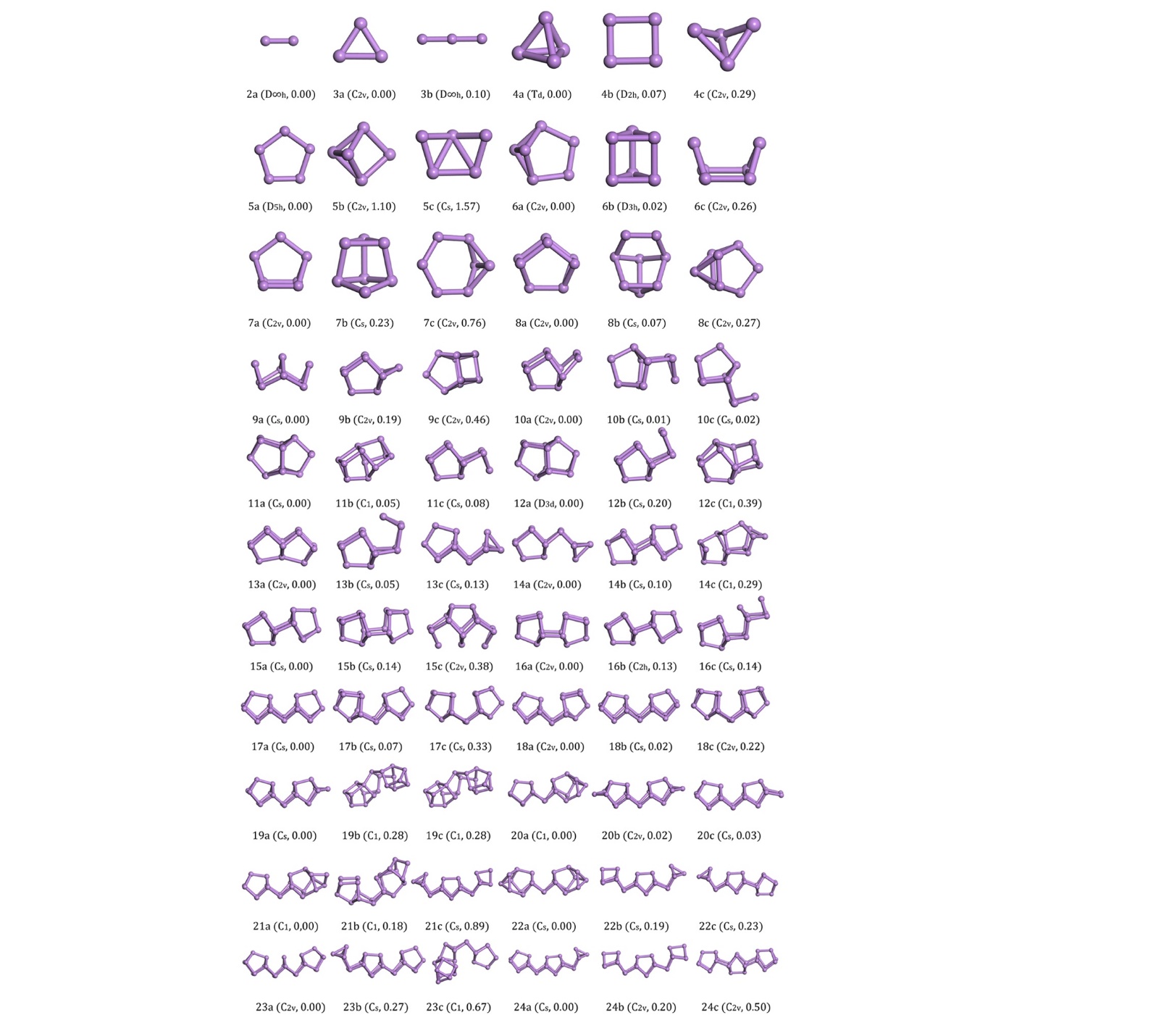


**Figure S3**. Low energy isomers of As^-^*_n_* (*n*=2–24) anionic clusters.
